# Inversion of Biological Strategies in Engineering Technology: A Case Study of the Underwater Soft Robot

**DOI:** 10.3390/biomimetics10060362

**Published:** 2025-06-03

**Authors:** Siqing Chen, He Xu, Xueyu Zhang, Tian Jiang, Zhen Ma

**Affiliations:** College of Electromechanical Engineering, Harbin Engineering University, Harbin 150001, China; bigfogfrog@hrbeu.edu.cn (S.C.);

**Keywords:** biological design, strategy inversion, soft robot

## Abstract

Bio-inspired design, a paradigm-shifting methodology that translates evolutionary mechanisms into engineering solutions, has established itself as a cornerstone for pioneering innovation in multifaceted technological systems. Despite its promise, the inherent complexity of biological systems and interdisciplinary knowledge gaps hinder the effective translation of biological principles into practical engineering solutions. This study introduces a structured framework integrating large language models (LLMs) with a function–behavior–characteristic–environment (F-B-C-E) paradigm to systematize biomimetic design processes. We propose a standardized F-B-C-E knowledge model to formalize biological strategy representations, coupled with a BERT-based pipeline for automated inversion of biological strategies into engineering applications. To optimize strategy selection, a hybrid decision-making methodology combining VIKOR multi-criteria analysis and rank correlation is developed. The framework’s functional robustness is validated via aquatic robotic system implementations, wherein three biomimetic propulsion modalities—oscillatory caudal propulsion, pulsed hydrodynamic thrust generation, and autonomous peristaltic locomotion—demonstrate quantifiable enhancements in locomotion efficiency and environmental adaptability metrics. These results underscore the robustness of the proposed inversion methodology in resolving intricate engineering problems through systematic biomimetic translation.

## 1. Introduction

Bio-inspired design addresses technical challenges by harnessing biological functions and principles [1,2,3,4], while also serving as a vital inspiration source for product innovation [5]. The core process of biomimetic inspired design can be divided into four progressive stages: problem definition, biological prototype screening, principle extraction, and engineering technology transformation [6]. This paradigm is essentially a cross-domain knowledge reconstruction process, utilizing existing biological characteristics, behaviors, and functions to correspond to features, behaviors, and similar functions in engineering, with the key being the efficiency of knowledge mapping between biological systems and engineering systems [7].

A fundamental challenge in contemporary research arises from the irreducible complexity of biological systems, which obstructs the extraction of actionable design principles, while existing engineering paradigms remain ill-equipped with systematic approaches to operationalize biological strategies within technological frameworks. The biological knowledge system is the foundation of biomimetic design, and solving engineering problems involves analogizing biological strategies [8]. Researchers with a biological background can explain the operational rules of natural systems well but lack knowledge reserves for engineering problems [9]. Engineers working in this field commonly encounter systemic barriers in identifying biological strategies, constrained by the professional barriers of the biological terminology system and the technical limitations of interdisciplinary knowledge expression [8,9]. Therefore, constructing an intelligent matching mechanism between biological characteristics and engineering parameters and improving the technical processes for screening biological prototypes and converting engineering technologies are important research directions for enhancing the effectiveness of biomimetic design.

In biomimetic design, the key steps lie in two points: (1) finding biological strategies corresponding to engineering problems (biological strategy mapping) and (2) expressing biological characteristics and behaviors in an engineering form (biological strategy inversion). In the mapping process, researchers analogize engineering problems to biological ones based on engineering requirements, seeking biological prototypes for solutions. This represents knowledge transfer from engineering to biology. In the inversion process, biological strategies from these prototypes are transformed into practical engineering strategies, representing knowledge transfer from biology to engineering. Biological strategy mapping is the foundation of biomimetic design, and it is crucial. The process involves systematically deconstructing the functional advantages formed by organisms during evolution using biological methods, establishing a cross-domain mapping relationship between biological systems and engineering requirements, and applying reverse engineering principles to convert biological strategies into implementable technical solutions. Current academic achievements focus on the digital representation of biological knowledge and morphological imitation, with insufficient attention paid to the integration mechanisms at the level of complex systems. Biomimetic engineering design falls within the realm of fuzzy decision-making scenarios, where descriptions of biological strategies mostly belong to natural language corpora [10].

Existing bionic design research often relies on experience-driven approaches, lacking a systematic knowledge framework and automated tools. This paper proposes a bionic design framework that integrates knowledge modeling, text analysis, and decision optimization. The core contributions include the function–behavior–characteristics in environment knowledge model (F-B-Cs in E knowledge model), standardizing the description of biological strategy functions (function), behaviors (behavior), characteristics (characteristic), and environmental associations (environment) as well as text-driven strategy transformations, which involve multi-label classification based on GPT models and engineering knowledge-based corrections to achieve automated mapping of biological strategies. Also presented is a hybrid multi-criteria decision-making method combining VIKOR with rank correlation analysis to balance designer preferences and objective metrics and select the optimal engineering strategy.

## 2. Related Works

The advantage of bio-inspired design lies in its ability to draw inspiration from biological mechanisms and strategies to develop innovative solutions [11]. This stems primarily from nature’s provision of abundant biological solutions to various engineering challenges [3]. However, a significant gap persists between existing biological strategy knowledge systems and the domains familiar to engineers [12]. One of the key objectives of BID is precisely to bridge this knowledge divide between biology and engineering [12].

Currently, numerous bio-inspired design methodologies have been extensively studied to facilitate the translation of natural principles into practical applications, such as Bio-TRIZ [13], biological functional modeling [14], and biomimetic design methods [15]. However, to the authors’ knowledge, existing research has provided limited systematic or automated assistance for engineers in utilizing biological strategies during engineering design and experimentation. Biomimicry, as a complement to the classical methods of innovative engineering, has enriched the diverse design of engineering solutions. In the process of bionic strategy inversion, solution generation based on functional modeling and solution generation based on conflict resolution are two mainstream solutions from biological prototype to engineering implementation [16]. These two dimensions correspond to the functional decomposition of the biological system and the reconciliation of contradictions, respectively.

Nagel et al. [17] proposed a biologically inspired dual-drive design framework, establishing the theoretical foundation for cross-domain knowledge transfer through problem-driven (engineering problem–biological solution mapping) and solution-driven (new biological discoveries–engineering migration) bidirectional pathways. Hancock et al. [18] introduced an evolution-driven design method, abstracting biological characteristics into “product genes” and simulating biological evolution through structural–function mapping and genetic algorithms to achieve autonomous optimization of components in biomimetic composite materials. Wiltgen et al. [19], on the other hand, introduced Case-Based Reasoning (CBR) technology, building a biological knowledge base that includes cases such as bat sonar and spider silk mechanics, using feature matching algorithms to shorten the generation cycle of engineering solutions.

Contradiction-driven design methodologies in bio-functional engineering systematize conflict resolution through a structured workflow encompassing conflict identification, cross-domain modeling, intelligent resolution, and functional validation. Current implementations leverage TRIZ contradiction matrices and quality function deployment (QFD) to detect technical incompatibilities arising from mismatches between biological traits and engineering specifications, and further operations are carried out using corresponding solutions from the contradiction matrix [20]. Similar to these methods, Zhao et al. [21] addressed the technical conflict between noise reduction and aerodynamic performance in the trailing edge serrated structure of wind turbine blades by developing a multi-disciplinary optimization-based aerodynamic–structural collaborative design method, creating an optimized design platform that balances noise reduction and aerodynamic efficiency. This ultimately resulted in a serrated airfoil with a 1.9% increase in lift-to-drag ratio, a 32.5% increase in lift coefficient, and reduced noise; Zuo et al. [22] tackled the physical conflict of flexible AC electrochromic devices struggling to achieve real-time wide-range color tunability and mechanical stability by developing a double-layer stacked luminescent structure based on dielectric difference regulation, optimizing the dielectric matrix material to enhance the device’s mechanical robustness and simulate biological camouflage and visual communication functions. The essence of this process is to achieve a transformation from contradictory opposition to collaborative innovation through the balance of biodiversity advantages and engineering constraints.

## 3. Inversion Methods of Biological Strategies

Biological adaptation strategies represent evolutionary optimizations refined through natural selection to address environmental challenges. In contrast, biomimetic design reframes engineering problems within biological paradigms, employing analogous evolutionary solutions for technological innovation. This systematic translation of biological principles into engineering contexts constitutes a conceptual inversion of natural adaptation mechanisms—termed “reverse biomimetics”—where biological strategies are recontextualized to solve human-defined technological constraints. While the screening of biological prototypes can identify numerous potential strategies, their effective implementation in engineering practice necessitates further exploration and validation. It should be noted that biological strategies primarily address issues within biological systems, whereas engineering problems often involve distinct complexities and constraints. Thus, converting biological strategies into engineering strategies, assessing their applicability in engineering, and selecting optimal approaches represent key focuses within biomimetic design research.

**Figure 1:**
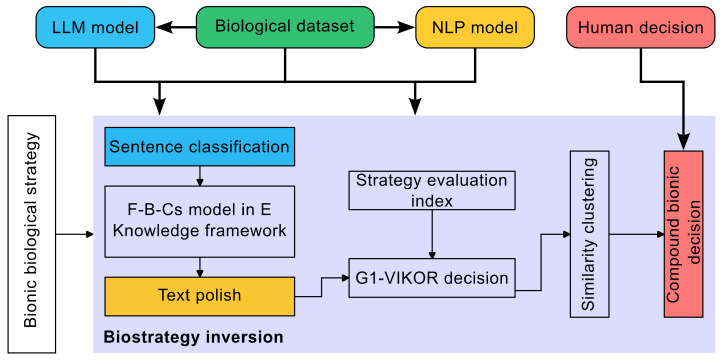
Bionic design process based on engineering mapping and strategy inversion.

The inversion process of biological strategies in this study is shown in Figure 1. The primary workflow encompasses the design of a knowledge framework, an inversion process aided by language models, and human evaluation and screening of the inversion outcomes. The knowledge framework serves as the foundation, offering a systematic approach to connect biological strategies with engineering requirements. LLM is deployed to streamline the inversion workflow, optimizing both the precision and efficacy of converting biological principles into engineering implementations. Human domain knowledge remains indispensable for critically evaluating and iteratively refining these outputs, thereby guaranteeing their functional viability and contextual relevance to real-world engineering challenges.

### 3.1. Knowledge Framework Design

In research on the inversion of biological strategies into engineering technology, a systematic and structured knowledge system is vital. It enhances designers’ understanding of biological mechanisms and principles. Complete text descriptions are not conducive to researchers’ summarization, communication, and computer batch processing. A comprehensive system can improve interaction efficiency between designers and computational tools, enhance knowledge sharing and reusability, and boost design process intelligence.

Current mainstream structured knowledge models include cognitive, causal, and functional models, with features listed in Table 1. In engineering, knowledge text is crucial for design reasoning, and biological strategy text must follow this framework. Under a unified logical architecture, switching framework instances can preliminarily invert biological strategies into engineering technology, requiring the knowledge text framework to be logically compatible with both fields.

Functional knowledge modeling formalizes biological systems by constructing a causal feature–behavior–function framework that maps biological capabilities to engineering objectives and technical specifications. This structured representation of biological principles aligns evolutionary mechanisms with engineered system requirements through systematic correspondence. Furthermore, environmental constraints in biological contexts are analogously translated into operational parameters for engineering applications.

**Figure 2:**
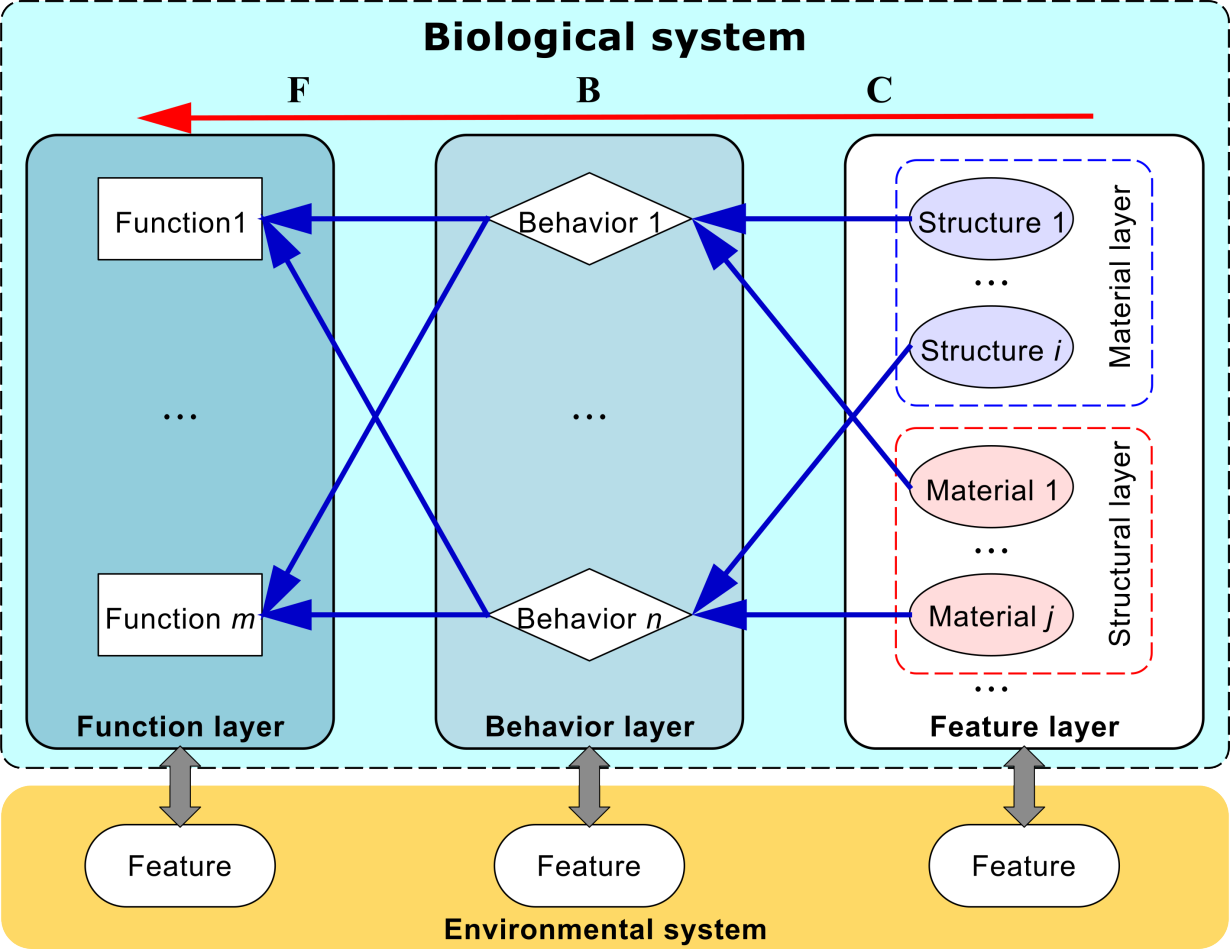
Knowledge framework based on characteristics–behaviors–functions in environmental systems.

This study constructs a knowledge framework connecting engineering and biology, explaining causal chains of instance behaviors in specific environmental contexts, as shown in Figure 2. The biological system in the model has three levels: function (F), behavior (B), and characteristic (C). Driven by environmental properties, biological strategies form material and structural characteristics to address environmental constraints and achieve functions. These strategies can also influence and alter the environment to suit their functions and behaviors.

F (function) refers to the purpose of biological systems and serves as the foundation for organizing and indexing biological strategies. In terms of description methods, it is composed of three elements: part of speech description (verb + noun), flow transformation description (involving energy, material, and signal flows), and state transformation description (from initial to final state).

B (behavior) describes the operational mechanisms of systems to achieve their functions. It is composed of single behavior description (ordered combination of different object functions) and cause–effect relationship description (cause + conjunction + effect).

C (characteristic) indicates the composition of systems, such as biological structures and material properties. It is composed of noun phrases, which include a main object and a relative clause for detailed description.

E (environment) consists of external factors that influence system operation. It is composed of input flow (environmental impact on biological instances) and output flow (impact of biological instances on the environment).

Elements like function, behavior, characteristic, and environment in the knowledge representation model are interconnected. However, current bionic texts usually have a single design path, linking all behaviors to one function. Although one behavior can correspond to multiple functions or vice versa, the text model should focus on a single function for inversion.

Thus, all bionic design texts and knowledge in this paper are designed as a model path combining single functions, single behaviors, and multiple characteristics under specific environments (F-B-Cs model in E). The causal relationships of behavior explain its causes and purposes, which are often ignored in text inversion. Multiple behavior processes can be handled in parallel in synchronous processing. From a scientific perspective, the “F-B-Cs model in E” model path is rational and feasible.

### 3.2. Inversion Process Assisted by Language Models

The translation of biological strategy texts into engineering frameworks requires systematizing biological knowledge through a structured representation schema. This formalized framework explicitly delineates functional objectives, behavioral patterns, characteristic attributes, and environmental domains within biological adaptation mechanisms. By transforming biological strategy texts into a standardized structure, not only does it help designers systematically organize their design ideas, but it also further enhances the accuracy of language processing dominated by commercial models. Therefore, the first step in biomimetic feature transformation is to systematically classify the originally scattered biological strategy texts into four dimensions: function, behavior, characteristic, and environment, and output them in a standardized format.

**Figure 3:**
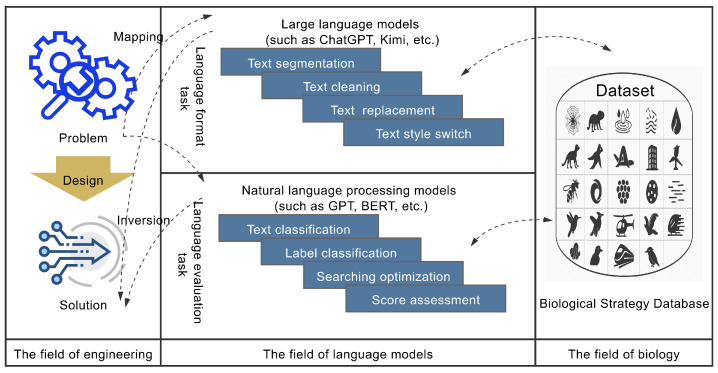
The approach of language models involved in bio-inspired design.

Both knowledge framework conversion and subsequent inversion processes involve handling biological and engineering texts. To boost bionic design intelligence, this paper presents a language model-assisted method for bionic inversion text processing, as shown in Figure 3. This approach elevates text summarization and knowledge routing in bionic design by harnessing the semantic integration capabilities of large language models (LLMs). Simultaneously, it improves the reliability of biomimetic text classification through task-specific NLP models optimized for precision in domain-relevant pattern recognition.

**Figure 4:**
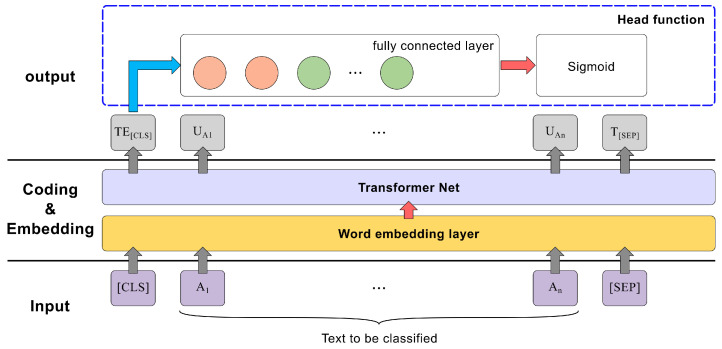
Structure of the label classification model.

Language is complex and diverse, and sentences often contain informationwith multiple aspects. Current commercial LLMs are ineffective and uneconomical for long text processing. To reduce their load, traditional language processing models are used to define the scope of text processing for LLMs. Given the multi-aspect nature of sentence information, the text processing task should be set as multi-label classification, allowing sentences to belong to multiple labels. When using the BERT model to compute label probabilities, the original BERT model architecture is adjusted; the input is set to single sentences, and the output adds a fully connected layer and a classification layer for precise label assignment, as shown in Figure 4. As labels in the classification task are not mutually exclusive, the Sigmoid function is chosen for the final classification layer. The BERT model is solely utilized to delineate the text’s scope, thereby regulating the LLM’s output and alleviating its processing load. This step constitutes the preprocessing phase in achieving text automation.

The basic data for text label classification is from a biological strategy library. After data cleaning and preprocessing, sentences are extracted from different texts using sentence symbols as training samples. The 18,888 bionic sentences screened from relevant documents are first input into the LLM. The model acts as a biologist and engineer to generate initial labels. Then, sentences and labels are organized into sets of 100 data points each. Random sampling of 3% is carried out for manual review. This continues until all statements and labels pass expert review, with no below-standard items found.

Considering the richness and complexity of language, data augmentation is applied to the text content. Sentences are randomly replaced by LLMs in certain proportions, combined with post review tags to form samples. Negative samples are not needed for the label classification task. In sample design, the sample size is controlled at 10,000, with a ratio of 8:2 for real to augmented samples to ensure balance and relevance.

The model parameter settings are shown in Table 2, and its training results are shown in Table 3.

With the BERT model’s assistance, each sentence in the bionic strategy corpus is labeled. These labels cover four aspects: function, behavior, characteristics, and environment. Note that a single text can have multiple labels. Based on these labels, bionic texts are categorized into four classes from longer texts. With the help of LLMs, these categories are systematically summarized and analyzed according to the previously mentioned biological knowledge structure framework. Researchers use LLMs to replace the subjects of biological strategies in texts and adjust the engineering knowledge framework based on part of speech structures and engineering logic.

**Figure 5:**
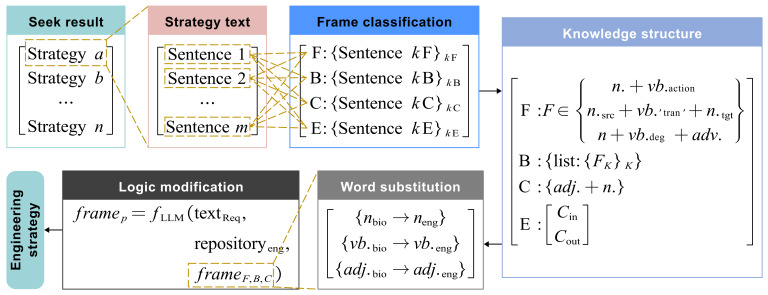
Bionic text conversion based on F-B-Cs model in E knowledge framework.

During the formative phase of model development, engineering prototypes may exhibit conceptual discrepancies in structural coherence and suboptimal lexical parameterization that deviate from domain-specific conventions. Crucially, large language models (LLMs) demonstrate the capability to address such issues through dynamic calibration mechanisms rooted in their multi-layered knowledge repositories. By leveraging context-aware optimization algorithms, these systems autonomously rectify semantic misalignments in technical descriptors while reinforcing logical interdependencies within the model architecture. This knowledge-driven rectification process not only ensures adherence to disciplinary nomenclature but also systematically enhances the operational fidelity of engineered systems. This ensures engineering solutions match the standard ones in the knowledge base and meet the designers’ objective theoretical logic, promoting the preliminary transition of biological strategies into engineering applications, as shown in Figure 5.

### 3.3. Process of Human Decision Making

In a specific engineering context, multiple biological strategies are selected from a biological strategy library, each possibly containing several relevant features. Thus, conducting coupled analysis, refinement, and generalization of these strategies and exploring their internal relationships and mutual influences are crucial steps in biomimetic design. Due to the targeted nature of biomimetic text descriptions, each biological strategy is treated as a primitive, serving as a basic element of biomimetic design. In engineering practice, multiple strategy primitives are often integrated to achieve the same function. Within the selected biological strategy set, similar, unrelated or even mutually exclusive behavior patterns may be observed, yet they all lead to the same function. Consequently, three major design paradigms exist in biomimetic design: imitative design based on a single biological primitive; coupled biomimetic design integrating multiple biological primitives; and subtractive design based on specific biological principles.

A multi-guided design framework involves two scenarios. First, different behaviors from various creatures achieve the same function, optimizing it through diverse actions. Second, different behaviors from various creatures guide different yet complementary functions, synergistically enhancing overall functional efficiency. When multiple creatures exhibit similar behaviors to achieve the same function, overlapping design resources may occur. In such cases, designers need to exercise caution, possibly implementing subtractive design on some strategies to ensure functional design implementation.

The key to distinguishing different design solutions lies in the degree of similarity between functions and behaviors. Given the unified format of inverted functions and behaviors, this paper uses the similarity estimation mentioned earlier to determine the possibility of multi-bionic design. The condition for two biological primitives to meet multi-coupled design requirements is that their functions are similar but their behaviors are unrelated. By leveraging LLMs to convert comparative texts into keywords and inputting them into an NLP-based relevance calculation model, the one-way relevance between two texts can be obtained. Thus, the NLP model can be seen as a function that converts two texts into a numerical value. Based on this value and by setting a distance threshold, texts can be clustered by comparing them within the knowledge framework. Bionic combinations of different types can be classified according to the following clustering rules:

(1) $$S_{\mathrm{MCB}}=\left\{S_{i}|S_{i}\in S_{S},K_{S,B}>k_{B},K_{S,F}>k_{F}\right\}$$

(2) $$\left\{\begin{array}{c} K_{S,B}=\Sigma_{i}\Sigma_{j}\frac{1-f_{\mathrm{NLP}}\left(f_{\mathrm{LLM}}\left(text_{Bj}\right),\;\;{\mathrm{text}}_{Bi}\right)}{\Sigma_{i}\Sigma_{j}H_{k,i,j}}\\ K_{S,F}=\Sigma_{i}\Sigma_{j}\frac{f_{\mathrm{NLP}}\left(f_{\mathrm{LLM}}\left(text_{Fj}\right),\;\;{\mathrm{text}}_{Fi}\right)}{\Sigma_{i}\Sigma_{j}H_{k,i,j}} \end{array}\right.$$

where $S_{\mathrm{MCB}}$ represents the coupling biomimetic strategy; $S_{S}$ represents the candidate strategies selected from the biological strategy library; $K_{S,B}$ represents the clustering score of strategy *S* in the behavior framework; $K_{S,F}$ represents the clustering score of strategy *S* in the function framework; $H_{k,i,j}$ represents the hypersphere unit function for similarity analysis; and $k_{B}$ and $k_{F}$ are the behavioral threshold and functional threshold.

Through biomimetic inversion, we can build a set of engineering strategies and use text clustering tech to derive biomimetic strategy sets. Despite using hybrid NLP and knowledge-based resources to meet practical engineering needs, the language model’s probabilistic nature and fragmentary output limit its grasp of design intent and engineering feasibility. Thus, final strategy selection relies on designers’ professional judgment.

Strategy evaluation, a multi-criterion decision making problem, involves designers adjusting weights based on preferences and priorities, driving innovation. After comparing common methods, this study uses the VIKOR method for comprehensive strategy evaluation.

When examining various bio strategies, their diverse features necessitate a systematic index system to capture their uniqueness. The evaluation index granularity must balance between being too broad (causing information loss and designer misjudgment) and too narrow (increasing complexity and technical difficulty, reducing system stability and operability). Based on the strategy library framework and general cost engineering standards, this study divides strategy evaluation indices into two levels. The first includes validity and feasibility. The second breaks validity into functional, behavioral, and characteristic fit; feasibility covers environmental adaptability, reliability, and economic tolerance.

Per the VIKOR method, the evaluation model has two alternatives, each with three indices. The evaluation values ($a_{ij}$) construct an initial matrix as follows:

(3) $$A\left\{a_{ij}\right\}=\left[\begin{array}{cccc} a_{11} & a_{12} & \cdots & a_{1n}\\ a_{21} & a_{22} & \cdots & a_{2n}\\ \vdots & \vdots & \ddots & \vdots \\ a_{m1} & a_{m2} & \cdots & a_{mn} \end{array}\right].$$

The decision making indicators in the text are subjectively scored by the designer for each item. To eliminate the impact of different attributes and dimensions of the indicators, normalization is needed. The normalization method is shown in Equation (4), and the final evaluation matrix (A) is obtained as shown in Equation (5).

(4) $$x_{ij}=\left\{\begin{array}{c} a_{ij}/\sqrt{\Sigma_{i}a_{ij}^{2}},\;\mathrm{Indicator}\;j\;\mathrm{belongs}\;\mathrm{to}\;\mathrm{positive}\;\mathrm{gain}\\ \frac{1}{a_{ij}}/\sqrt{\Sigma_{i}{\left(\frac{1}{a_{ij}}\right)}^{2}},\;\mathrm{Indicator}\;j\;\mathrm{belongs}\;\mathrm{to}\;\mathrm{negative}\;\mathrm{gain} \end{array}\right.,$$

(5) $$A=\left[\begin{array}{cccc} x_{11} & x_{12} & \cdots & x_{1n}\\ x_{21} & x_{22} & \cdots & x_{2n}\\ \vdots & \vdots & \ddots & \vdots \\ x_{m1} & x_{m2} & \cdots & x_{mn} \end{array}\right].$$

where $x_{ij}$ represents the numerical value of the indicator after orthogonalization.

Rank correlations of evaluation indicators can be obtained by ranking the importance of all indicators, which is expressed as

(6) $$x_{1}^{*}≻x_{2}^{*}≻\cdots ≻x_{j}^{*}$$

The reasonable determination of the ratio between the importance of adjacent evaluation indicators and the importance of the evaluation indicators themselves is designated $r_{j}$, and its value can be obtained by referring to Table 4. The weights (ω) of all indicators can be calculated using the following equation:

(7) $$r_{j}=\omega_{j-1}/\omega_{j},$$

(8) $$\omega_{m}={\left(1+\overset{2\to m}{\Sigma_{k}}\overset{k\to m}{\Pi_{z}}r_{z}\right)}^{-1}.$$

After calculating the weights, decision making can be carried out using the VIKOR method. The group effect $\left(s_{i}\right)$ and individual regret $\left(R_{i}\right)$ values of the decision-making objects need to be calculated using Equations (9) to (11), and the compromise value $Q_{i}$ of each evaluated object can be determined. The group effect and individual regret values are intermediate variables. The final decision is determined based on the compromise value.

(9) $$s_{i}=\overset{1\to n}{\Sigma_{j}}\frac{\omega_{j}\left(x_{j}^{+}-x_{ij}\right)}{x_{j}^{+}-x_{j}^{-}},$$

(10) $$R_{i}=\max_{j}\left\{\frac{\omega_{j}\left(x_{j}^{+}-x_{ij}\right)}{x_{j}^{+}-x_{j}^{-}}\right\},$$

(11) $$Q_{i}=\varepsilon \frac{s_{i}-s^{-}}{s^{+}-s^{-}}+\left(1-\varepsilon \right)\frac{R_{i}-R^{-}}{R^{+}-R^{-}}.$$

In the strategy’s decision-making process, strategies are sorted in ascending order based on $s_{i}$, $R_{i}$, and $Q_{i}$, with the first engineering strategy being the optimum. If the following two criteria are met, strategies are ranked by $Q_{i}$, where a smaller $Q_{i}$ indicates a better solution. If the second criterion is not satisfied, the compromise solution set is $\left\{A_{1},A_{2}\right\}$. If the top-ranked solution does not meet Criterion 1 but satisfies Criterion 2, the compromise solution set is $\left\{A_{1},A_{2},\ldots ,A_{m}\right\}$, with *m* determined by $Q\left(A_{2}\right)Q\left(A_{1}\right)\geq 1/\left(m-1\right)$.

Criterion 1: The acceptable advantage criterion is $Q\left(A_{2}\right)Q\left(A_{1}\right)\geq 1/\left(m-1\right)$, where $A_{1}$ and $A_{2}$ are the optimal and suboptimal objects obtained from each scheme’s ranking by $Q_{i}$.

Criterion 2: The acceptable stability criterion is that $A_{1}$ is the optimal target based on the ranking by $s_{i}$ or $Q_{i}$.

**Figure 6:**
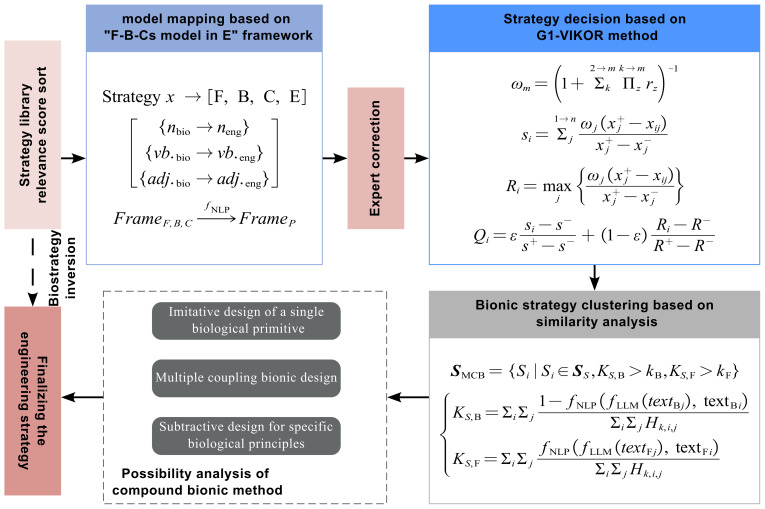
The total inversion process of biological strategy in engineering technology.

Thus, the inversion process of the entire biomimetic strategy is shown in Figure 6. NLP is used to classify relevant texts into knowledge framework labels based on biological strategies. LLMs then summarize the corresponding text into the knowledge framework structure and transform it into initial engineering strategies via part of speech substitution. Based on the evaluation of human engineering strategy effectiveness, similar strategies are clustered to obtain feasible engineering strategies.

## 4. Case Study

Traditional underwater rigid robots have numerous limitations. Their rigid materials with high elastic modulus result in a hard appearance, poor environmental adaptability, and a tendency to cause harm when coming into contact with marine life; traditional jet propulsion methods also create significant disturbances to the underwater environment, affecting the normal lives of marine organisms. Furthermore, due to the inclusion of mechanical components such as control, transmission, and actuation parts, traditional underwater rigid robots are relatively large in size, making operations in narrow underwater spaces difficult. Compared with traditional underwater robots, underwater soft robots exhibit better environmental adaptability and flexibility, enabling safer contact with marine life, reducing interference with the underwater environment, and their compact design allows them to operate effectively in narrow spaces. Soft robots are a hot topic in bionic design. Therefore, this paper takes the movement of underwater soft robots as a case study with which to demonstrate the design of three different movement strategies.

**Figure 7:**
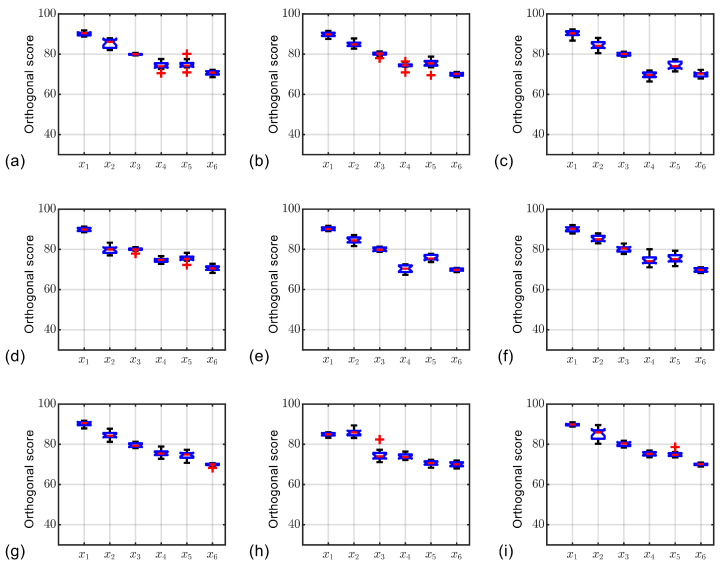
The evaluation scores of the strategies from (**a**–**i**) No. 1 to No. 9.

**Figure 8:**
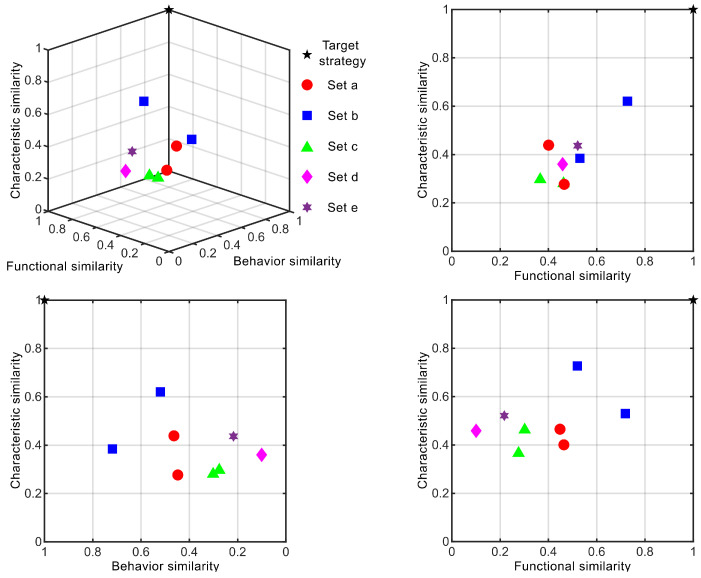
The spatial distribution of biomimetics strategies’ clustering in the motion design strategy of the underwater robot.

In this section, we evaluate the effectiveness of biomimetic inversion through various case studies. We examine the different movement strategies of soft robots, all derived selected from our prior biomimetic research [23]. The biological cases underlying the soft robot movement strategies are illustrated in Table 5. The weight distribution of the determination strategy is shown in Table 6. Through the preliminary inversion (Figure 4), the clustering process of strategies is shown in Figure 7, calculated by Equations (1) and (2). As shown in Figure 8, all strategies have been manually screened to eliminate unsuitable ones. Evaluation during the design process is based on the compromise value of each strategy, calculated by Equations (3)–(11). Similar strategies are merged through strategy clustering, and unsuitable ones are eliminated via evaluation. Each strategy undergoes an inversion process due to the convenience of LLM, which will be detailed in the subsequent text.

The strategies screened out from the biological strategy library are as follows:

(1) Tail Oscillation Propulsion Strategy

The biomechanical principles of this strategy follow the law of conservation of momentum, generating reaction thrust through periodic flow field disturbances. This propulsion mechanism shares similarities with the vortex shedding observed in fluid dynamics.

(2) Exteroskeleton Friction-Driven Propulsion Strategy

This strategy set includes two types of microbial motion patterns that have been revised by experts. Both utilize the viscoelastic interaction between surface appendages (like cilia or pseudopodia) and the substrate to generate thrust. Despite traditional morphological clustering, from a biomechanical perspective, they exhibit non-inertia dominated characteristics in low-Reynolds-number fluid environments, differing significantly in energy utilization from macro scale organisms.

(3) Fluid Ejection Propulsion Strategy

Characterized by a specialized fluid storage and ejection system, this strategy is exemplified by the pulsatile jet propulsion of jellyfish and the coordinated mantle funnel movements of cephalopods like squid. Both follow the momentum jet principle for incompressible fluids, with propulsion efficiency assessable via the product of jet mass flow rate and exit velocity.

(4) Segmented Coordination Peristaltic Propulsion Strategy

Based on the rhythmic movement of neuromuscular systems, this strategy generates traveling waves through phase differences between adjacent segments. It achieves net displacement by dynamically modulating the friction coefficient at the contact interface. Unlike the centralized drive Strategy Set a, this strategy employs a distributed drive mechanism with energetically autonomous units.

(5) Buoyancy-Modulated Displacement Strategy

This innovative strategy leverages asymmetric buoyancy distribution, adjusting the spatial relationship between the center of buoyancy and the center of gravity via an internal buoyancy regulation system. This propulsion mechanism exploits controlled hydrostatic differentials to generate directional displacement, establishing a bio-derived paradigm for mechanism-free propulsion systems with demonstrated efficacy in low-turbulence aquatic environments.

In this paper, three strategies with higher manual scores are selected for inversion and implements the design in the engineering field.

### 4.1. Tail Swing Propulsion Strategy

Fish propel themselves through body and tail fin undulations, creating lateral waves that form reverse Karman vortices for propulsion [24]. Cetaceans like whales and dolphins use tail fluke oscillations, leveraging high-Reynolds-number flow fields for efficient movement. Their tail fins’ flexible material gradients allow them to bear high fluid loads while adjusting to large attack angles and enhancing thrust via leading edge vortex attachment. The knowledge framework of this biological strategy is presented in Equation (12).

(12) $$S_{\mathrm{bio}}^{\mathrm{swim}}=\left[\begin{array}{c} \begin{array}{cc} F & \mathrm{Provide}\;\mathrm{propulsion} \end{array}\\ \begin{array}{cc} B & \left[\begin{array}{c} \mathrm{Generating}\;\mathrm{flexion}\;\mathrm{through}\;\mathrm{tail}\;\mathrm{undulation}\\ \mathrm{Alternating}\;\mathrm{musculature}\;\mathrm{contractions}\\ \mathrm{Converting}\;\mathrm{spinal}\;\mathrm{elastic}\;\mathrm{energy}\\ \mathrm{Delaying}\;\mathrm{flow}\;\mathrm{separation}\;\mathrm{with}\;\mathrm{keel}\;\mathrm{structure} \end{array}\right] \end{array}\\ \begin{array}{cc} C & \left[\begin{array}{c} \mathrm{Peduncular}\;\mathrm{elastic}\;\mathrm{ligament}\;\mathrm{system}\\ \mathrm{Polymorphic}\;\mathrm{caudal}\;\mathrm{fin}\;\mathrm{architectures}\\ \mathrm{Scale}\;\mathrm{microgrooved}\;\mathrm{highspeed}\;\mathrm{species}\\ \mathrm{hydrodynamic}\;\mathrm{keel}\;\mathrm{morpho} \end{array}\right] \end{array} \end{array}\right]$$

The biological principles governing aquatic locomotion have profoundly informed bionic robotic design. While fish generate thrust through undulatory body waves, cetaceans employ oscillatory caudal fin movements—a propulsion mechanism demonstrating reduced sensitivity to primary body geometry limitations. This distinction has led engineers to favor the caudal fin oscillation paradigm, as its operational practicality offers greater implementation feasibility in mechanical systems.

**Figure 9:**
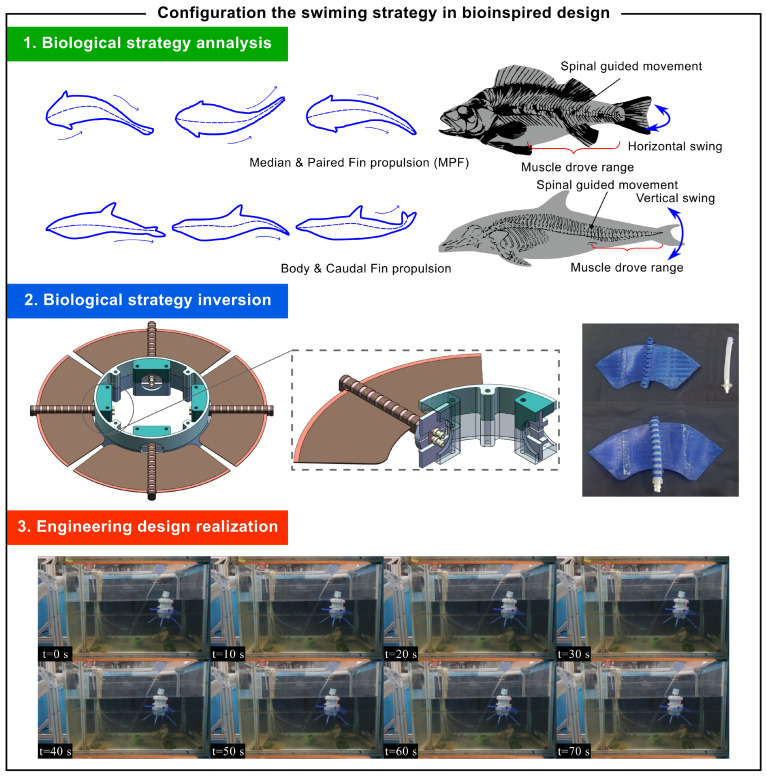
Case: tail swing propulsion strategy.

In bionic robot design, the driver is inspired by the fish muscle spine system, as shown in Figure 9. The power for tail oscillation comes from red muscle fiber contraction, a biomechanical property that can be replicated using a specific driver. Optimizing the cross-sectional thickness of the airbag structure can enhance the driver’s bending performance. Among different wall thickness schemes, the 0.5 mm wall thickness driver is chosen for its effective response under high pressure and lower manufacturing risks. The knowledge framework of the engineering strategy is presented in (13). The specific design work is described in detail in the reference [25].

(13) $$S_{\mathrm{base}}^{\mathrm{swim}}=\Gamma \left(S_{e}^{1}⊕S_{e}^{7}⊕S_{e}^{9}\right)=\left[\begin{array}{c} \begin{array}{cc} F & \mathrm{Trust}\;\mathrm{Vector}\;\mathrm{Control} \end{array}\\ \begin{array}{cc} B & \left[\begin{array}{c} \mathrm{Driving}\;\mathrm{flexible}\;\mathrm{structure}\\ \mathrm{The}\;\mathrm{internal}\;\mathrm{structure}\\ \mathrm{Propagating}\;\mathrm{pressure}\;\mathrm{wave}\\ \mathrm{Generate}\;\mathrm{directional}\;\mathrm{flow}\\ \mathrm{Differential}\;\mathrm{steering} \end{array}\right] \end{array}\\ \begin{array}{cc} C & \left[\begin{array}{c} \mathrm{Flexible}\;\mathrm{accessory}\;\mathrm{structure}\\ \mathrm{Hydrodynamic}\;\mathrm{surface}\\ \mathrm{Modular}\;\mathrm{tap}\;\mathrm{structure} \end{array}\right] \end{array} \end{array}\right]$$

Three-dimensional printing technology enables the integrated molding of drivers and wearable exoskeletons. Adjusting the exoskeleton’s gap parameters can effectively control the driver’s bending degree of freedom and deformation extent. Experimental results show that a 3 mm gap exoskeleton structure performs well in practice.

The mobile module design is inspired by biological tail structures, simplifying control for multi-directional movement. Experiments confirm that the untethered soft robot demonstrates good mobility in a simulated underwater environment, achieving a maximum average speed of 1.42 mm/s. These findings provide a significant theoretical and practical foundation for bionic robot design.

### 4.2. Fluid Injection Propulsion Strategy

Cephalopods’ jet propulsion system exhibits unique biomechanical optimization [26]. Their specialized mantle, circular muscles, and directional nozzle work together to store and rapidly release elastic energy, generating high internal pressure for efficient movement. This biological mechanism maintains laminar flow in the jet core region, minimizing turbulent energy loss and demonstrating superior propulsion efficiency. The knowledge framework of this biological strategy is presented in Equation (14).

(14) $$S_{\mathrm{bio}}^{\mathrm{prop}}=\left[\begin{array}{c} \begin{array}{cc} F & \mathrm{Provide}\;\mathrm{fluid}\;\mathrm{propulsion} \end{array}\\ \begin{array}{cc} B & \left[\begin{array}{c} \mathrm{Rhythmic}\;\mathrm{contraction}\;\mathrm{of}\;\mathrm{the}\;\mathrm{bell}\\ \mathrm{Expelling}\;\mathrm{water}\;\mathrm{backward}\;\mathrm{to}\;\mathrm{generate}\;\mathrm{thrust}\\ \mathrm{Assisting}\;\mathrm{bell}\;\mathrm{recovery}\;\mathrm{with}\;\mathrm{radial}\;\mathrm{muscles}\\ \mathrm{Utilizing}\;\mathrm{elastic}\;\mathrm{recoil}\;\mathrm{force}\;\mathrm{for}\;\mathrm{energy}\;\mathrm{recovery} \end{array}\right] \end{array}\\ \begin{array}{cc} C & \left[\begin{array}{c} \mathrm{Gelatinous}\;\mathrm{bell}\;\mathrm{with}\;\mathrm{water}\;\mathrm{content}\\ \mathrm{Orthogonal}\;\mathrm{grid}\;\mathrm{structure}\;\mathrm{of}\;\mathrm{circular}\;\mathrm{and}\;\mathrm{radial}\;\mathrm{muscles}\\ \mathrm{Elastic}\;\mathrm{folds}\;\mathrm{on}\;\mathrm{bell}\;\mathrm{margin}\\ \mathrm{Collagen}\;\mathrm{fibers}\;\mathrm{for}\;\mathrm{energy}\;\mathrm{storage}\; \end{array}\right] \end{array} \end{array}\right]$$

**Figure 10:**
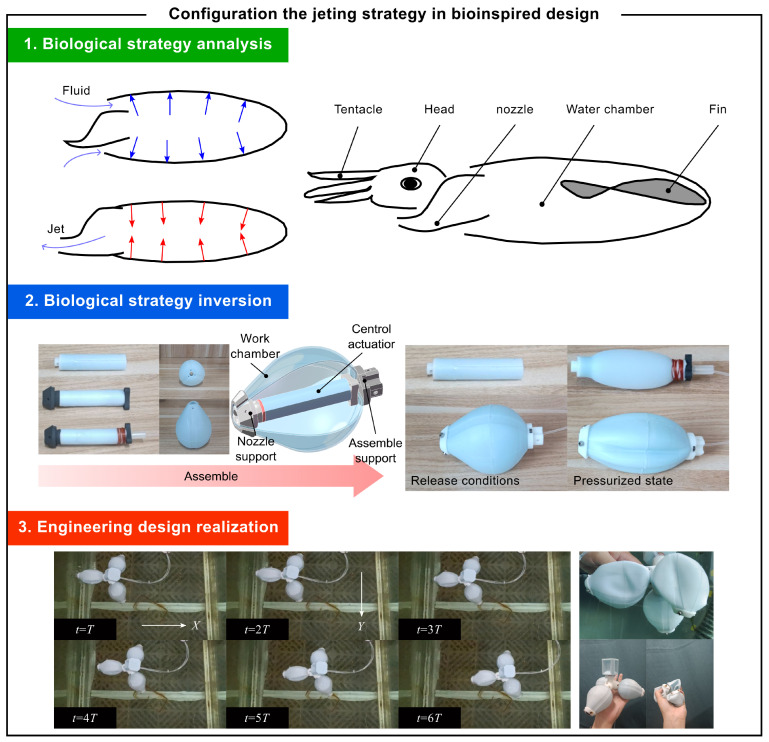
Case: fluid injection propulsion strategy.

In engineering terms, this biological mechanism inspires soft robot design, as shown in Figure 10. The elastic energy storage of biological pressure chambers can be analogous to pre-stressed balloon structures in robots. By applying pressure to the air sacs of soft actuators, the active contraction mechanism of biological muscles is replicated. This approach enables the design of robots capable of controlled and efficient jet propulsion. The knowledge framework of the engineering strategy is presented in (15). The specific design work is described in detail in the reference [27].

(15) $$S_{\mathrm{base}}^{\mathrm{prop}}=\Gamma \left(S_{e}^{2}⊕S_{e}^{3}\right)=\left[\begin{array}{c} \begin{array}{cc} F & \mathrm{Provide}\;\mathrm{underwater}\;\mathrm{thrust} \end{array}\\ \begin{array}{cc} B & \left[\begin{array}{c} \mathrm{Rapid}\;\mathrm{fluid}\;\mathrm{removal}\\ \mathrm{Rotary}\;\mathrm{jet}\;\mathrm{chase}\\ \mathrm{Recovery}\;\mathrm{by}\;\mathrm{elastic}\;\mathrm{potential}\;\mathrm{energy}\\ \mathrm{Use}\;\mathrm{external}\;\mathrm{structures}\;\mathrm{to}\;\mathrm{motion} \end{array}\right] \end{array}\\ \begin{array}{cc} C & \left[\begin{array}{c} \mathrm{Drive}\;\mathrm{mechanism}\;\mathrm{extrusion}\\ \mathrm{Nozzle}\;\mathrm{based}\;\mathrm{on}\;\mathrm{rigid}\;\mathrm{support}\\ \mathrm{Exterior}\;\mathrm{diamond}-\mathrm{shaped}\;\mathrm{structure}\\ \mathrm{Elastic}\;\mathrm{cavity}\; \end{array}\right] \end{array} \end{array}\right]$$

Experimental results highlight the effectiveness of this design. The untethered soft robot demonstrates remarkable mobility in simulated underwater environments, achieving a maximum ascent speed of 98 mm/s and a descent speed of 52 mm/s. Its ability to move horizontally under the jet unit’s propulsion and slowly land on the water bottom confirms the practical viability of this bio-inspired approach.

### 4.3. Autonomic Peristalsis Strategy

In nature, inchworms crawl efficiently using a segment coordination mechanism [28]. Their movement involves anchoring via micro hooks on their posterior prolegs, redistributing hydraulic pressure through muscle contractions, and extending their bodies through sequential muscle activation. This process allows them to achieve forward movement with each cycle of contraction and extension. The knowledge framework of this biological strategy is presented in Equation (16).

(16) $$S_{\mathrm{bio}}^{\mathrm{cra}}=\left[\begin{array}{c} \begin{array}{cc} F & \mathrm{Enable}\;\mathrm{crawling}\;\mathrm{locomotion} \end{array}\\ \begin{array}{cc} B & \left[\begin{array}{c} \mathrm{Contract}\;\mathrm{circular}\;\mathrm{muscles}\;\mathrm{to}\;\mathrm{detach}\;\mathrm{from}\;\mathrm{substrate}\\ \mathrm{Relax}\;\mathrm{radial}\;\mathrm{muscles}\;\mathrm{to}\;\mathrm{generate}\;\mathrm{negative}\;\mathrm{pressure}\\ \mathrm{Expel}\;\mathrm{water}\;\mathrm{through}\;\mathrm{contraction}\;\mathrm{to}\;\mathrm{propel}\;\mathrm{forward}\\ \mathrm{Transmit}\;\mathrm{contraction}\;\mathrm{waves}\;\mathrm{for}\;\mathrm{coordinated}\;\mathrm{movement} \end{array}\right] \end{array}\\ \begin{array}{cc} C & \left[\begin{array}{c} \mathrm{Radially}\;\mathrm{symmetrical}\;\mathrm{body}\;\mathrm{structure}\\ \mathrm{Bilayer}\;\mathrm{body}\;\mathrm{wall}\;\mathrm{with}\;\mathrm{mesoglea}\;\mathrm{rich}\;\mathrm{in}\;\mathrm{collagen}\\ \mathrm{Presence}\;\mathrm{of}\;\mathrm{specialized}\;\mathrm{muscle}\;\mathrm{fibers}\;\mathrm{and}\;\mathrm{gland}\;\mathrm{cells} \end{array}\right] \end{array} \end{array}\right]$$

**Figure 11:**
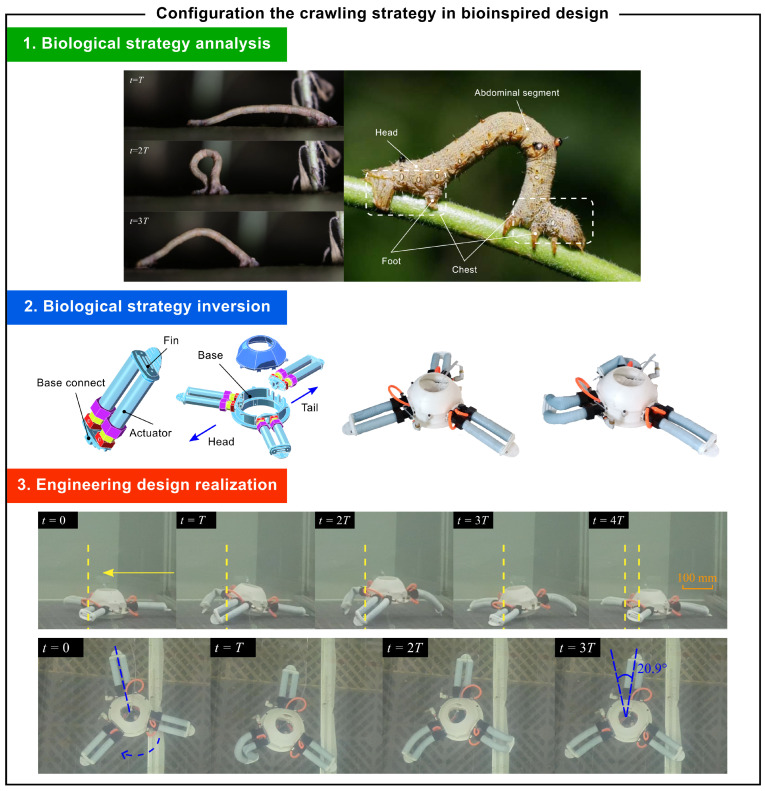
Case: crawling strategy based on autonomic peristalsis.

In engineering applications, this biological mechanism inspires the design of soft robots, as shown in Figure 11. The bio-inspired design employs a driver array to mimic the muscle movement distribution of inchworms. The drivers feature silicone tubes with sealed ends, where silicone is molded around embedded fibers in a 3D-printed mold. This design enhances radial stiffness while constraining axial expansion, enabling the robot to bend in a controlled manner and achieve complex movements. The knowledge framework of the engineering strategy is presented in 17. The specific design work is described in detail in the reference [29].

(17) $$S_{\mathrm{base}}^{\mathrm{cra}}=\Gamma \left(S_{e}^{4}⊕S_{e}^{5}\right)=\left[\begin{array}{c} \begin{array}{cc} F & \mathrm{Achieve}\;\mathrm{crawling} \end{array}\\ \begin{array}{cc} B & \left[\begin{array}{c} \mathrm{Coordinate}\;\mathrm{muscle}\;\mathrm{movement}\\ \mathrm{Shrink}\;\mathrm{driver}\\ \mathrm{Release}\;\mathrm{the}\;\mathrm{drive}\;\mathrm{from}\;\mathrm{the}\;\mathrm{base}\\ \mathrm{Transmitted}\;\mathrm{contraction}\;\mathrm{wave} \end{array}\right] \end{array}\\ \begin{array}{cc} C & \left[\begin{array}{c} \mathrm{Radial}\;\mathrm{symmetric}\;\mathrm{structure}\\ \mathrm{Contact}\;\mathrm{friction}\;\mathrm{control}\\ \mathrm{Still}\;\mathrm{water}\;\mathrm{skeleton}\;\mathrm{system} \end{array}\right] \end{array} \end{array}\right]$$

The experimental robot demonstrates inchworm-like crawling through its triangular driver layout, enabling multi-directional movement on the seafloor. The robot’s motion aligns with the inchworm inspired model, showing efficient locomotion even on low-friction surfaces. These experiments validate the effectiveness of the biomimetic design approach in creating soft robots capable of controlled and adaptive movement.

## 5. Conclusions

Identifying key biological strategies for engineering applications is crucial in biomimetic design. This paper addresses engineering problem mapping and biological strategy inversion. By combining traditional NLP models with knowledge graphs, it enables rapid matching and optimization of biological and engineering strategies within the F-B-C-E knowledge framework, establishing technical routes for subsequent designs. A knowledge framework linking engineering and biology has been built, allowing systematic analysis of biological prototypes across functional, behavioral, characteristic, and environmental dimensions. Analogies are drawn using large language models and engineering logic from knowledge bases. Finally, a hybrid multi-criterion decision making method is applied to evaluate and rank the mapped engineering strategies. Multiple evaluation indicators are considered, such as functional suitability, behavioral and characteristic alignment, environmental transferability, reliability, and economic tolerance, to identify optimal engineering strategies.

This paper’s main contributions are as follows:

(1) Using LLMs and traditional NLP models to rapidly process bionic texts;
(2) Demonstrating optimal engineering strategy recommendations through multidimensional decision making in strategy selection;
(3) Validating the inversion method’s effectiveness via three application cases. The results show that the inversion method enhances the efficiency and feasibility of bionic design.

## Figures and Tables

**Table 1:** The advantages and disadvantages of different structured knowledge models.

Model Type	Representative Model	Focus	Limitations
Cognitive Model	SBF Model	Demonstrating the internal operation mechanism of biological principles; Describing the transformation of input/output states, state transfer paths, and causality; Elaborating the functional characteristics, behavioral patterns, and structural layout of instances.	Lacks representation of environmental knowledge; Limits the mapping and analogy of cross-domain knowledge;
Causal Model	SAPPhIRE Model	Constructing system state transfer paths based on cause–effect relationships between the internal structures and functions of biological instances;	Fine-grained partitioning increases the complexity of knowledge modeling; Insufficient clarity in describing functional and structural information.
Functional Model	FCKM Model	Focusing on the expression at the system function level; Revealing the functional topology and reasoning process within biological instances.	The model granularity is relatively coarse, and completeness is lacking; Lacks in-depth representation of causality and behavioral mechanisms within biological instances.

**Table 2:** Parameter setting for BERT model.

Parameter	Value
Learning rate	$1.2\times 10^{-5}$
Epoch	17
Batch size	64
Optimizer	Adam

**Table 3:** Evaluation index in the process of the BERT model training.

Precision	Recall	Accuracy	F1-Score
0.85	0.77	0.89	0.81

**Table 4:** The reference value used to determine the importance of the adjacent indicator.

$r_{c}$	Definition
1	Indicator $X_{j-1}$ is of equal importance to Indicator $X_{j}$.
1.2	Indicator $X_{j-1}$ is slightly more important than Indicator $X_{j}$.
1.4	Indicator $X_{j-1}$ is significantly more important than Indicator $X_{j}$.
1.6	Indicator $X_{j-1}$ is much more important than Indicator $X_{j}$.
1.8	Indicator $X_{j-1}$ is extremely more important than Indicator $X_{j}$.

**Table 5:** Biomimetics strategies for motions of underwater robot.

NO.	Biomimetic Strategies	Rank	*Q*	Set
1	Marine Fish Propulsion Optimized by Tail Undulation and Fluid Dynamics	1	0.0635	b
2	Jellyfish Movement Optimized by Pulsatile Propulsion and Fluid Sensing	3	0.2143	c
3	Squid Movement Driven by Biological Jet Propulsion	8	0.7932	c
4	Cnidarians Move via Hydrostatic Skeleton and Muscle Coordination	7	0.4880	a
5	Copepod Propulsion via Asynchronous Cilia and Microstructure Integration	6	0.3476	a
6	Echinoderms Move by Water Vascular and Collagen–Muscle Integration	5	0.2528	e
7	Marine Mammals’ Swimming with Fluid Dynamics and Energy Storage	4	0.2518	b
8	Physalia physalis Drifts via Asymmetrical Float and Tentacle Coordination	9	1.0851	d
9	Octopus Buoyancy Control via Gill-Heart and Mantle Muscle Coordination	2	0.1568	b

**Table 6:** The weight distribution of the engineering strategy.

$\omega_{1}$	$\omega_{2}$	$\omega_{3}$	$\omega_{4}$	$\omega_{5}$	$\omega_{6}$
0.2048	0.2458	0.2048	0.0790	0.0948	0.1707

## Data Availability

The datasets generated during and or analyzed during the current study are not publicly available as the data also forms part of an ongoing study.
